# *Clitoria ternatea* and *Moringa oleifera* extracts reduce endothelial activation and intimal thickening in diet-induced atherosclerosis

**DOI:** 10.17305/bb.2026.14277

**Published:** 2026-05-26

**Authors:** Karnirius Harefa, Ahmad Hafizullah Ritonga, Riri Safitri, Barita Aritonang, Rahmad Gurusinga, Sri Wulan, Beny Irawan, Rostime Hermayerni Simanullang

**Affiliations:** 1 Institut Kesehatan Medistra Lubuk Pakam, Lubuk Pakam, Deli Serdang, Indonesia; 2 Department of Biomedical Science, Universitas Murni Teguh, Medan, Indonesia

**Keywords:** *Clitoria ternatea*, *Moringa oleifera*, atherosclerosis, anti-inflammatory agents, tunica intima

## Abstract

Atherosclerosis is a chronic inflammatory vascular disease characterized by endothelial activation and progressive thickening of the tunica intima. This study aimed to evaluate the effects of combined *Clitoria ternatea* and *Moringa oleifera* (CT-MO) extracts, designated as CT-MO, on endothelial activation markers and tunica intima thickness in male Wistar rats fed a high-fat and high-cholesterol (HFHC) diet. Phytochemical screening and ultraviolet-visible (UV-Vis) spectrophotometry were conducted to determine the optimal extract formulation, which was subsequently administered *in vivo* at doses of 200, 400, and 600 mg/kg body weight for 45 days. Serum levels of vascular cell adhesion molecule-1 (VCAM-1), intercellular adhesion molecule-1 (ICAM-1), interleukin-6 (IL-6), and cluster of differentiation 40 (CD40) were measured using enzyme-linked immunosorbent assay (ELISA), while tunica intima thickness was assessed histologically. Correlation and exploratory multivariate regression analyses were conducted to evaluate associations between inflammatory biomarkers and vascular structural remodeling. The CT-MO (25:75) combination demonstrated the highest flavonoid content (79.10 mg/g). The HFHC diet significantly increased inflammatory biomarkers and tunica intima thickness (12.22 µm). Treatment with CT-MO extract significantly reduced VCAM-1, ICAM-1, IL-6, and CD40 levels in a dose-dependent manner (*P*< 0.05). Correlation analysis demonstrated significant positive associations between inflammatory biomarkers and tunica intima thickness, while exploratory multivariate analysis identified VCAM-1 as the biomarker most strongly associated with vascular structural remodeling. The highest dose (600 mg/kg) exhibited the most pronounced effect, with biomarker levels not significantly different from healthy controls and tunica intima thickness reduced to 3.42 µm. These findings suggest that the combined CT-MO extract may attenuate endothelial activation and vascular structural remodeling associated with early atherosclerosis.

## Introduction

Atherosclerosis remains the leading cause of cardiovascular diseases, significantly contributing to global morbidity and mortality [1–3]. It is characterized by chronic inflammation of the arterial wall, resulting from endothelial dysfunction, lipid accumulation, immune cell recruitment, and the formation of vascular plaques [4–8]. A key molecular mechanism underlying the initiation and progression of atherosclerosis is the overexpression of endothelial adhesion molecules, notably Vascular Cell Adhesion Molecule-1 (VCAM-1) [9–11] and Intercellular Adhesion Molecule-1 (ICAM-1) [12–14]. These molecules facilitate leukocyte adhesion and transmigration into the intima, thereby exacerbating local vascular inflammation [15–17]. Additionally, pro-inflammatory mediators such as Interleukin-6 (IL-6) and CD40 play crucial roles in sustaining immune activation associated with the progression of vascular inflammation [18–20]. Elevated expression levels of these biomarkers are considered indicators of the severity of vascular inflammation and correlate with inflammatory activity during atherosclerosis development [21, 22].

In addition to molecular inflammation, structural remodeling of the arterial wall is a critical aspect of atherosclerosis progression. The tunica intima, as the innermost arterial layer, serves as the primary site for lipid deposition and leukocyte infiltration during early atherogenesis [20, 23]. Persistent endothelial activation increases vascular permeability and promotes the accumulation of inflammatory cells within the intimal layer, leading to progressive thickening of the tunica intima. This structural alteration reflects vascular remodeling and is recognized as a key morphological hallmark of atherosclerosis severity [24, 25]. Consequently, evaluating the thickness of the tunica intima provides complementary histological evidence to circulating inflammatory biomarkers, allowing for a more comprehensive assessment of vascular injury. These findings indicate that atherosclerosis involves both inflammatory endothelial activation and structural vascular remodeling. Therefore, effective therapeutic strategies should target not only circulating inflammatory mediators but also the structural changes within the arterial wall [20, 24].

Current pharmacological management of atherosclerosis primarily relies on synthetic agents such as statins, fibrates, and antiplatelet drugs, which focus on lipid reduction and inflammation control. However, long-term use of these agents is often associated with adverse effects, including hepatotoxicity, myopathy, and drug resistance [5, 26–29]. Consequently, recent research has shifted toward exploring natural compounds that exhibit comparable anti-inflammatory and antioxidant effects but have lower toxicity profiles [30–32].

Among medicinal plants, *Clitoria ternatea* (CT), also known as the butterfly pea flower, and *Moringa oleifera* (MO) leaves have gained attention for their potent bioactive compounds. CT is rich in anthocyanins, flavonoids, and phenolics, which have been reported to suppress IL-6 and ICAM-1 expression by mitigating oxidative stress and inhibiting nuclear factor kappa B (NF-κB) signaling [15, 22, 33]. Similarly, MO contains isothiocyanates and polyphenols with strong anti-inflammatory potential, shown to reduce VCAM-1, IL-6, and CD40 expression in hyperlipidemic and inflammatory animal models [34–36].

Several studies have reinforced the potential of these two plants as antioxidant and anti-inflammatory agents relevant to the atherosclerotic process. Widowati et al. [33] reported that CT extract exhibited antidiabetic effects through antioxidant and anti-inflammatory activities that reduced oxidative stress and improved lipid profiles in diabetic and dyslipidemic rat models, conditions closely associated with atherogenesis. Saengnak et al. [37] demonstrated that CT extract suppressed the angiotensin II (Ang II)–NADPH oxidase 4 (NOX4) oxidative stress pathway in hypertensive rats, thereby reducing endothelial injury and the risk of atherosclerosis. Meanwhile, Elghandour et al. [34] found that nano-encapsulated MO extract exhibited significant metabolic and antioxidant activities, potentially improving oxidative and inflammatory status associated with atherosclerosis development.

Despite these promising findings, limited evidence exists regarding the combined effects of CT and MO extracts on both endothelial activation and tunica intima remodeling in diet-induced atherosclerosis. Most previous investigations have focused on single-extract interventions and circulating biomarkers, without integrating molecular inflammatory markers and structural vascular assessments. Furthermore, existing studies have not comprehensively addressed whether combined phytochemical interventions can concurrently impact both endothelial dysfunction and vascular remodeling [20, 35, 36]. This gap underscores the necessity for studies evaluating whether a combined formulation can simultaneously modulate endothelial activation and mitigate tunica intima thickening. The proposed conceptual framework of this study posits that phytochemical compounds present in *Clitoria ternatea–Moringa oleifera* (CT-MO) extract may attenuate inflammatory signaling associated with endothelial activation, thereby reducing adhesion molecule expression and limiting vascular remodeling as reflected by tunica intima thickening. Therefore, this study aims to evaluate the effects of a combined CT-MO extract on endothelial activation and tunica intima thickening in a diet-induced early atherosclerosis model, focusing on key inflammatory biomarkers (VCAM-1, ICAM-1, IL-6, and CD40) as indicators of vascular inflammation severity. It is hypothesized that the combined extract may exert concurrent anti-inflammatory effects and reduce vascular structural damage.

## Materials and methods

### Experimental design and animals

This study was conducted as a controlled post-test experimental investigation to evaluate the effects of CT, MO, and combined CT-MO extracts on diet-induced early atherosclerosis in male Wistar rats. The experiment was carried out from July to October 2025 at the Organic Chemistry and Pharmacology Laboratories, Faculty of Pharmacy, Institut Kesehatan Medistra Lubuk Pakam, Indonesia.

A total of thirty-five male Wistar rats (*Rattus norvegicus* L.), aged 3–4 months and weighing 180–200 g, were included. The animals were acclimatized for 7 days prior to the experiment under standard laboratory conditions (temperature 22—25 ^∘^C, 12 h light/dark cycle, and free access to food and water). The animals were randomly assigned to seven groups (*n* ═ 5 per group) and treated for 45 days. The sample size (*n* ═ 5 per group) was selected based on commonly accepted practices and previous exploratory biomarker-based animal studies evaluating inflammatory biomarkers and histological outcomes. No formal a priori power analysis was performed, as this study was designed as an exploratory preliminary investigation. Randomization was performed using a computer-generated random number method, and investigators responsible for outcome assessment were blinded to group allocation to minimize bias. Daily monitoring was conducted to record any physiological or behavioral variations. All treatments were administered orally via gastric gavage once daily. The negative and disease control groups also received oral gavage with equivalent volumes of 1% sodium carboxymethyl cellulose (Na-CMC) to maintain consistent handling conditions across groups. Details of the treatment schedule are presented in Table 1.

**Table 1:** Experimental animal treatment groups

**Group**	**Code**	**Treatment description**
Disease control	DC	Administered HFHC diet, PTU, and 1% Na-CMC.
CT-MO 200 mg/kg	CT-MO200	Administered HFHC diet, PTU, 1% Na-CMC, and CT-MO extract at a dosage of 200 mg/kg BW.
CT-MO 400 mg/kg	CT-MO400	Administered HFHC diet, PTU, 1% Na-CMC, and CT-MO extract at a dosage of 400 mg/kg BW.
CT-MO 600 mg/kg	CT-MO600	Administered HFHC diet, PTU, 1% Na-CMC, and CT-MO extract at a dosage of 600 mg/kg BW.
CT 600 mg/kg	CT600	Administered HFHC diet, PTU, 1% Na-CMC, and CT extract at a dosage of 600 mg/kg BW.
MO 600 mg/kg	MO600	Administered HFHC diet, PTU, 1% Na-CMC, and MO extract at a dosage of 600 mg/kg BW.
Negative control	NC	Administered standard diet and 1% Na-CMC via oral gavage.

Abbreviations: BW, body weight; CT, Clitoria ternatea; CT-MO, Clitoria ternatea–Moringa oleifera; DC, disease control; HFHC, high-fat and high-cholesterol; MO, Moringa oleifera; Na-CMC, sodium carboxymethyl cellulose; NC, negative control; PTU, propylthiouracil.

### Preparation of CT-MO extracts

CT flowers and MO leaves were washed and oven-dried at 40—50 ^∘^C until a constant weight was achieved. The dried materials were ground separately into fine powders and sieved through a 40-mesh screen to obtain a uniform particle size. For single-extract preparation, CT and MO powders were extracted individually. For combined (CT-MO) extracts, the powdered simplicia were blended before extraction to obtain formulations with CT:MO ratios of 75:25, 50:50, and 25:75 (w/w), with a total batch weight of 400 g for each formulation. Each batch was macerated in 96% ethanol at a solvent-to-material ratio of 5:1 (v/w) for 72 h at room temperature in the dark with intermittent stirring. The residual marc was re-macerated with fresh solvent for an additional 24 h. The combined filtrates were concentrated using a rotary evaporator at 40^∘^C. The extracts were stored at 4^∘^C until further analysis [15, 37]. The extraction yields of CT, MO, CT-MO (75:25), CT-MO (50:50), and CT-MO (25:75) extracts were approximately 12.4%, 24.1%, 16.8%, 17.5%, and 18.6%, respectively, calculated based on the dry weight of the starting powdered plant materials after solvent evaporation.

### Phytochemical screening of CT-MO extract

Phytochemical screening was conducted to detect flavonoids, phenolics, tannins, saponins, terpenoids, and alkaloids in CT, MO, and their combined extracts. Flavonoids were identified by the development of a yellow coloration following the reaction of the extract with magnesium powder and concentrated HCl. Phenolic compounds were qualitatively detected using ferric chloride (FeCl3), indicated by the formation of a green to dark green coloration. Tannins were evaluated using the FeCl3 test after dissolving the extract in hot distilled water, followed by cooling and filtration, where a blue-green coloration indicated the presence of tannins. Saponins were detected by the formation of stable foam after vigorous shaking of the extract with distilled water. Terpenoids were identified by the development of a reddish-brown coloration after reaction with glacial acetic acid and concentrated H2SO4. Alkaloids were confirmed by the formation of a brown precipitate following treatment with chloroform, concentrated H2SO4, and Wagner’s reagent. All phytochemical tests were performed using standard qualitative procedures as previously described [22, 38].

### Quantitative phytochemical analysis by ultraviolet-visible (UV-Vis) spectrophotometry

Preliminary phytochemical screening revealed the presence of phenolic compounds, tannins, and flavonoids. Quantitative analysis of total phenolic, tannin, and flavonoid contents in CT, MO, and CT-MO formulations was conducted using UV-Vis spectrophotometry. The total phenolic content was measured using the Folin-Ciocalteu method with gallic acid standards (20–100 ppm) at 765 nm. Tannin content was quantified using tannic acid standards (20–100 ppm) at 737 nm. Total flavonoid content was assessed using the aluminum chloride colorimetric method with quercetin standards (20–100 ppm) at 431 nm [39–41]. Calibration curves were established for each standard, and the coefficient of determination (R^2^) was calculated to evaluate linearity. All assays were performed in triplicate, and the results were expressed as mean ± standard deviation in mg gallic acid equivalent (GAE)/g extract, mg tannic acid equivalent (TAE)/g extract, and mg quercetin equivalent (QE)/g extract [39, 42]. The formulation with the highest flavonoid content was selected for subsequent *in vivo* evaluation.

### Preparation of a high-fat and high-cholesterol diet

Rats were fed *ad libitum* with a high-fat and high-cholesterol (HFHC) diet. This formulation comprised 10% beef fat, 1% pure cholesterol, and 0.2% cholic acid, which were thoroughly blended with the standard All Feed-2 diet (Central Proteinaprima Company, Indonesia) to achieve a uniform mixture. The mixture was finely ground and reformed into solid pellets to ensure consistency and facilitate feeding. The HFHC diet was prepared in 4 kg batches, each sufficient for approximately 11 days of feeding. Each batch included 400 g of beef fat, 40 g of pure cholesterol, 8 g of cholic acid, and 3,552 g of the standard All Feed-2 diet, totaling 4,000 g. This diet formulation was adapted from previously established atherosclerosis-inducing protocols [22, 43]. In this study, the HFHC diet was combined with propylthiouracil (PTU) to enhance the development of atherosclerotic features throughout the experimental period.

### PTU induction through drinking water administration

Rats were provided ad libitum access to drinking water containing PTU at a daily dosage of 1 mg/kg body weight (BW). Based on an average BW of 200 g, each rat required approximately 0.2 mg of PTU per day. PTU-containing drinking water was administered to the disease control (DC), CT-MO200, CT-MO400, CT-MO600, CT600, and MO600 groups, while the negative control (NC) group received standard drinking water without PTU. The PTU solution was freshly prepared daily for a total of 30 rats. A total of 6 mg of PTU was dissolved in 750 mL of drinking water, corresponding to an average consumption of approximately 25 mL per rat per day. The solution was provided to each cage to ensure consistent exposure across treatment groups. The combination of the HFHC diet and PTU administration was utilized to establish a reproducible atherosclerosis model. PTU induces a hypothyroid state, which is known to impair lipid metabolism, elevate serum cholesterol levels, and contribute to vascular dysfunction, thereby enhancing the atherogenic effects of the HFHC diet. This combined approach has been reported to facilitate more consistent and pronounced atherosclerotic changes within a shorter experimental duration compared to dietary induction alone [44, 45].

### Preparation of 1% Na-CMC solution

A 1% Na-CMC solution was prepared by dispersing 10 g of Na-CMC powder in 200 mL of distilled water. The mixture was heated and allowed to swell for approximately 15 min, followed by continuous stirring until a homogeneous solution was formed. Distilled water was then gradually added to achieve a final volume of 1000 mL [13, 22].

### Preparation and oral administration of CT-MO extract suspensions

Extract suspensions of CT, MO, and CT–MO (25:75, w/w) were prepared using 1% Na-CMC as the vehicle. Dose calculations were based on an average body weight of 200 g per rat. Body weight was measured weekly, and doses were adjusted accordingly to maintain accuracy. For the CT–MO treatment groups, the required daily doses per rat were 0.04 g, 0.08 g, and 0.12 g for the 200, 400, and 600 mg/kg BW doses, respectively. For practical administration, suspensions were prepared weekly for each group (*n* ═ 5 rats) to cover 7 consecutive days. Accordingly, total extract amounts of 1.4 g, 2.8 g, and 4.2 g were dissolved in 1% Na-CMC to a final volume of 35 mL for the respective doses. Prepared suspensions were stored at 4^∘^C and homogenized before administration to ensure stability and uniformity. Each rat received 1 mL of the suspension via oral gavage once daily according to its treatment group. For single-extract groups (CT600 and MO600), the same calculation was applied using a dose of 600 mg/kg BW (0.12 g per rat per day), with weekly preparation adjusted proportionally for five rats [15, 20].

### Biomarker measurement and histological analysis

Serum samples obtained from experimental rats were analyzed for atherosclerosis-associated biomarkers using a sandwich enzyme-linked immunosorbent assay (ELISA) with commercial kits from Elabscience (Texas, USA). Each target molecule (VCAM-1, ICAM-1, IL-6, and CD40) was quantified according to the manufacturer’s recommended protocol. All samples were analyzed in triplicate to ensure analytical reliability. The assays for soluble VCAM-1/CD106 (Cat. No. E-EL-R0910), ICAM-1/CD54 (Cat. No. E-EL-R0046), IL-6 (Cat. No. E-EL-R0015), and CD40 (Cat. No. E-EL-R1196) were conducted within their respective detection ranges as specified by the manufacturer. Following color development, absorbance was measured using a Bio-Rad microplate spectrophotometer (Model 680). For histological evaluation, thoracic aortas were carefully excised after the treatment period, rinsed with physiological saline, and fixed in 10% neutral-buffered formalin. Tissues were processed using standard paraffin-embedding techniques, sectioned at 4–5 µm thickness, and stained with hematoxylin and eosin (H&E). Histological measurements were performed by an independent observer blinded to treatment groups. Tunica intima thickness was measured using a light microscope equipped with an ocular micrometer, following previously described methods. Measurements were obtained at 400× magnification from five randomly selected fields per section, and the mean value for each animal was used for statistical analysis. Inflammatory biomarker levels were further analyzed concerning tunica intima thickness to explore associations between vascular inflammation and structural remodeling [20, 23].

### Ethical statement

All experimental procedures were approved by the Animal Research Ethics Committee (AREC), Faculty of Mathematics and Natural Sciences, Universitas Sumatera Utara (Approval No. 0601/KEPH-FMIPA/2025), and conducted in accordance with established guidelines for the care and use of laboratory animals. All procedures complied with relevant institutional and national guidelines for animal welfare.

### Statistical analysis

Data were expressed as mean ± standard deviation (SD). Statistical analyses were performed using Statistical Package for the Social Sciences (SPSS) software. Data normality and homogeneity of variance were assessed using the Shapiro–Wilk and Levene’s tests prior to analysis. Differences among groups were analyzed using one-way analysis of variance (ANOVA), followed by Tukey’s post hoc test for pairwise comparisons. Effect sizes were estimated using partial eta squared (η^2^). Pearson correlation analysis was conducted to evaluate associations between inflammatory biomarkers and tunica intima thickness. Exploratory multivariate linear regression analysis was performed using tunica intima thickness as the dependent variable and inflammatory biomarkers (VCAM-1, ICAM-1, IL-6, and CD40) as predictor variables. Ninety-five percent confidence intervals (95% CI) were calculated for regression coefficients where appropriate. A *P*-value < 0.05 was considered statistically significant. The statistical analyses in this exploratory study aimed to evaluate group differences and explore associations between inflammatory biomarkers and vascular structural remodeling [15].

## Results

### Phytochemical screening results of CT-MO extract

Phytochemical screening was conducted on single extracts of CT, MO, and combined CT-MO extracts. Qualitative analysis demonstrated the presence of flavonoids, phenolic compounds, and tannins in both single and combined extracts, while saponins, terpenoids, and alkaloids were not detected under the applied test conditions (Table 2). The presence of phenolic compounds and tannins was indicated by the development of green to blue–green coloration upon reaction with ferric chloride solution, while flavonoids were confirmed through the magnesium–hydrochloric acid reaction. No stable foam formation was observed in the saponin test, no reddish-brown coloration was detected in the terpenoid test, and no precipitate formation was noted in the alkaloid test. These results confirm the consistent presence of flavonoids, phenolic compounds, and tannins across all extracts, while saponins, terpenoids, and alkaloids were not detected under the experimental conditions.

**Table 2:** Phytochemical profile of CT, MO, and CT-MO extract

**Compound**	**Reagent**	**Observed result**	**CT**	**MO**	**CT-MO**
Flavonoids	Mg + concentrated HCl	Bright yellow	(+)	(+)	(+)
Tannins	5% FeCl_3_ solution	Blue-green	(+)	(+)	(+)
Phenolics	5% FeCl_3_ solution	Dark green	(+)	(+)	(+)
Saponins	Foam test	No stable foam	(--)	(--)	(--)
Terpenoids	Glacial acetic acid + concentrated H_2_SO_4_	No reddish-brown ring	(--)	(--)	(--)
Alkaloids	CHCl_3_ + conc. H_2_SO_4_ + Wagner’s reagent	No precipitate	(--)	(--)	(--)

Abbreviations: CHCl_3_, chloroform; CT, *Clitoria ternatea*; CT-MO, *Clitoria ternatea–Moringa oleifera*; FeCl_3_, ferric chloride; HCl, hydrochloric acid; H_2_SO_4_, sulfuric acid; Mg, magnesium; MO, *Moringa oleifera*.

**Table 3:** UV-Vis spectrophotometric quantification of tannin, phenolic, and flavonoid concentrations

**Sample**	**Tannin** **(mg TAE/g)**	**Phenolic** **(mg GAE/g)**	**Flavonoid** **(mg QE/g)**
CT extract	47.4217 ± 0.0640	40.1403 ± 0.1650	46.5158 ± 0.1557
MO extract	110.0473 ± 2.2840	91.9486 ± 0.0724	38.1785 ± 0.1544
Combined CT-MO extract (75:25)	53.3086 ± 0.1969	48.3677 ± 0.0301	44.3726 ± 0.1457
Combined CT-MO extract (50:50)	65.9243 ± 0.1727	55.7152 ± 0.0183	57.1121 ± 0.1142
Combined CT-MO extract (25:75)	78.1170 ± 0.1004	59.4907 ± 0.0138	79.1028 ± 0.1346

All values are expressed as the mean ± SD (*n* ═ 3). Abbreviations: CT, *Clitoria ternatea*; CT-MO, *Clitoria ternatea–Moringa oleifera*; GAE, gallic acid equivalent; MO, *Moringa oleifera*; QE, quercetin equivalent; SD, standard deviation; TAE, tannic acid equivalent; UV-Vis, ultraviolet-visible.

### Quantitative phytochemical profiling and selection of CT-MO (25:75)

The tannin, total phenolic, and flavonoid contents of CT, MO, and their combined CT–MO extracts were quantified using UV–Vis spectrophotometry following qualitative phytochemical screening (Table 3). Among the individual extracts, MO exhibited higher tannin and total phenolic contents compared to CT, while CT demonstrated greater flavonoid levels than MO. In the combined formulations, both tannin and total phenolic contents increased with higher proportions of MO. Notably, the CT–MO (25:75) formulation exhibited the highest flavonoid content (79.1028 mg QE/g), along with tannin (78.1170 mg TAE/g) and total phenolic (59.4907 mg GAE/g) levels in comparison to other formulations. Based on these findings, CT–MO (25:75, w/w) was selected for subsequent *in vivo* evaluation.

### Effects of CT-MO extract on VCAM-1 levels

**Figure 1.**
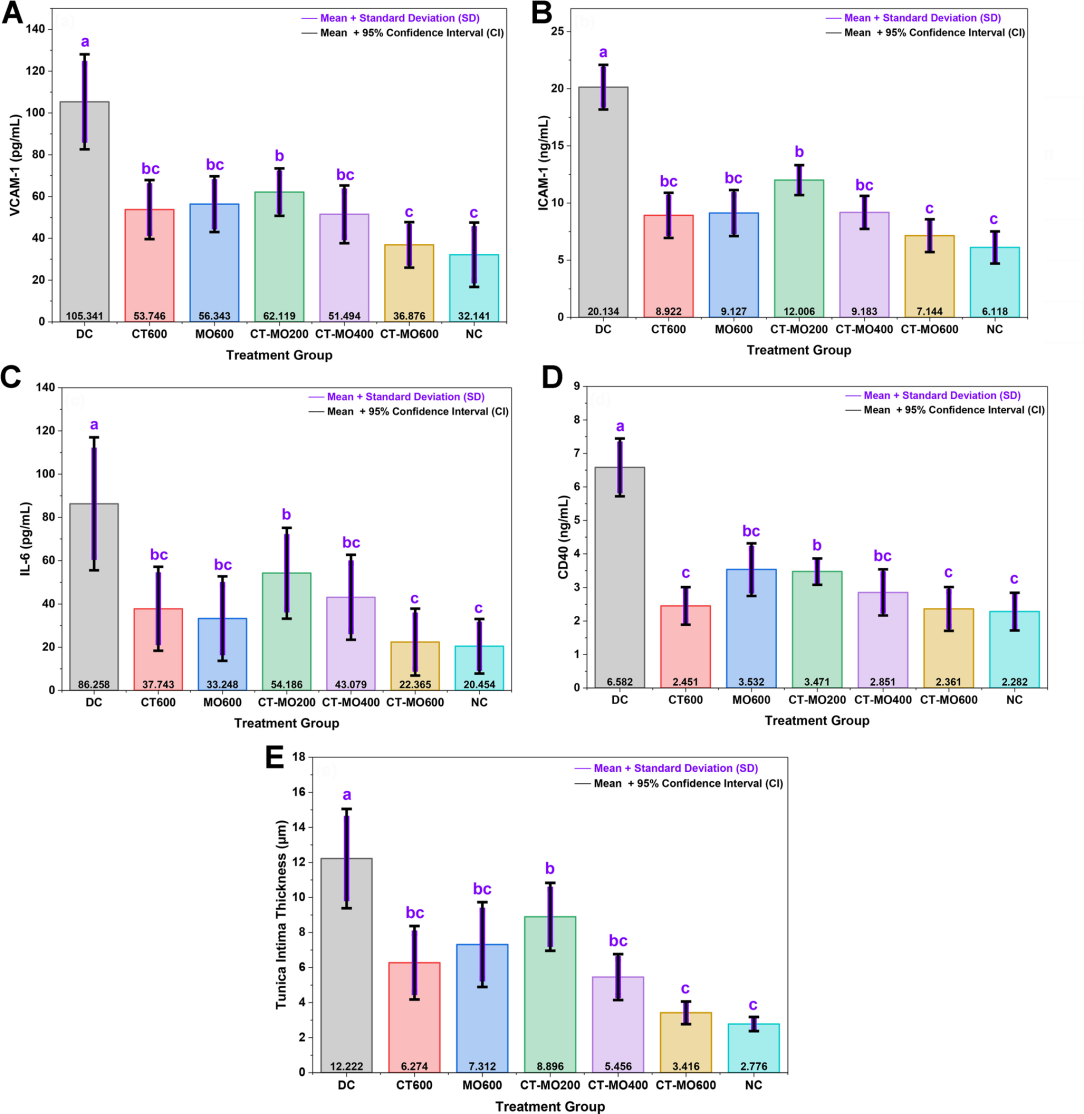
**Effects of combined ***Clitoria ternatea–Moringa oleifera*** extract on inflammatory biomarkers and tunica intima thickness in diet-induced early atherosclerosis.** Serum levels of (A) VCAM-1, (B) ICAM-1, (C) IL-6, and (D) CD40, together with (E) tunica intima thickness, were compared among experimental groups of male Wistar rats. The HFHC diet with PTU markedly increased inflammatory biomarker levels and tunica intima thickness in the DC group, whereas treatment with CT-MO extract reduced these parameters in a dose-dependent manner. The CT-MO600 group showed the greatest reduction, with biomarker levels and tunica intima thickness approaching those observed in the NC group. Data are presented as mean ± SD, with 95% CI shown as indicated in the figure (*n* ═ 5 per group). Different lowercase letters indicate statistically significant differences between groups, while groups sharing at least one letter are not significantly different according to one-way ANOVA followed by Tukey’s post hoc test (*P* < 0.05). Abbreviations: ANOVA, analysis of variance; CD40, cluster of differentiation 40; CI, confidence interval; CT, *Clitoria ternatea*; CT-MO, *Clitoria ternatea–Moringa oleifera*; DC, disease control; HFHC, high-fat and high-cholesterol; ICAM-1, intercellular adhesion molecule-1; IL-6, interleukin-6; MO, *Moringa oleifera*; NC, negative control; PTU, propylthiouracil; SD, standard deviation; VCAM-1, vascular cell adhesion molecule-1.

One-way ANOVA indicated significant differences in VCAM-1 levels among the experimental groups (*F* ═ 19.621, *P* < 0.001; η^2^ ═ 0.808), reflecting a substantial treatment effect (Table 4). Induction of a HFHC diet in the DC group significantly elevated VCAM-1 levels (105.34 pg/mL) compared to the NC group (32.14 pg/mL). Administration of both single and combined extracts effectively reduced this elevation (Figure 1A). Tukey’s honestly significant difference (HSD) test confirmed that all treatment groups exhibited a significant reduction in VCAM-1 levels relative to the DC group (*P* < 0.05). Single extracts of CT600 (53.74 pg/mL) and MO600 (56.34 pg/mL) demonstrated comparable efficacy. In contrast, the combined CT-MO extract exhibited a dose-dependent reduction, with the lowest VCAM-1 level recorded in the CT-MO600 group (36.87 pg/mL). Notably, Tukey’s analysis revealed no significant difference between the CT-MO600 and NC groups, indicating that VCAM-1 levels in the CT-MO600 group were statistically similar to those of the NC group (*P* > 0.05).

**Table 4:** One-way ANOVA of inflammatory biomarkers and tunica intima thickness

**Variable**	***F* value**	***P* value**	**Partial Eta squared (η^2^)**	**Interpretation**
VCAM-1	19.621	< 0.001	0.808	Very large effect
ICAM-1	60.404	< 0.001	0.928	Very large effect
IL-6	9.375	< 0.001	0.668	Large effect
CD40	40.476	< 0.001	0.897	Very large effect
Tunica intima	23.503	< 0.001	0.834	Very large effect

Abbreviations: ANOVA, analysis of variance; CD40, cluster of differentiation 40; ICAM-1, intercellular adhesion molecule-1; IL-6, interleukin-6; VCAM-1, vascular cell adhesion molecule-1.

### Effects of CT-MO extract on ICAM-1 levels

Significant differences in ICAM-1 levels were observed across groups (*F* ═ 60.404, *P* < 0.001; η^2^ ═ 0.928), indicating an exceptionally large treatment effect (Table 4). The HFHC diet resulted in a dramatic increase in ICAM-1 concentration in the DC group (20.13 ± 1.54 ng/mL) compared to the NC group (6.11 ± 1.31 ng/mL). Intervention with either single or combined extracts effectively mitigated this increase (Figure 1B). Tukey’s HSD test revealed that all treatment groups achieved significant reductions in ICAM-1 levels compared to the DC group (*P* < 0.05). Single treatments of CT600 (8.92 ± 1.57 ng/mL) and MO600 (9.12 ± 1.63 ng/mL) yielded comparable effects. Among the combined formulations, a clear dose-dependent improvement was evident. The lowest ICAM-1 level was noted in the CT-MO600 group (7.14 ± 1.22 ng/mL), with biomarker levels statistically comparable to those of healthy controls. This suggests that the highest combination dose was associated with a greater reduction in ICAM-1 levels, approaching baseline values.

### Effects of CT-MO extract on IL-6 levels

IL-6 levels varied significantly among groups (*F* ═ 9.375, *P* < 0.001; η^2^ ═ 0.668) (Table 4). The HFHC diet significantly elevated IL-6 levels in the DC group (86.25 ± 28.72 pg/mL) compared to the NC group (20.45 ± 14.41 pg/mL), but this inflammatory response was effectively attenuated by all extract treatments (Figure 1C). Tukey’s HSD test indicated that single extracts of CT600 (37.54 ± 18.34 pg/mL) and MO600 (33.24 ± 17.57 pg/mL) resulted in reductions, while the combined CT-MO extract exhibited a dose-dependent decrease in IL-6 levels. Notably, the CT-MO600 group (22.36 ± 14.58 pg/mL) achieved the most substantial reduction, with IL-6 levels statistically indistinguishable from those of the healthy NC group, indicating potential attenuation of systemic inflammatory responses to levels comparable to the NC group.

### Effects of CT-MO extract on CD40 levels

CD40 levels also exhibited significant differences among groups (*F* ═ 40.476, *P* < 0.001; η^2^ ═ 0.897) (Table 4). The HFHC diet in the DC group induced a significant increase in CD40 levels (6.58 ± 0.68 ng/mL) compared to the NC group (2.28 ± 0.43 ng/mL) (Figure 1D). All intervention groups successfully reduced this increase, and Tukey’s HSD test confirmed significant reductions across all treatments relative to the DC group (*P* < 0.05). While the combination groups exhibited a dose-dependent improvement from CT-MO200 (3.47 ± 0.31 ng/mL) to CT-MO400 (2.85 ± 0.59 ng/mL), only the CT-MO600 group (2.36 ± 0.52 ng/mL) reached levels indistinguishable from those of the healthy NC group. These findings suggest that the 600 mg/kg BW combination dose was associated with the most pronounced reduction in CD40 levels, approaching values comparable to the NC group.

### Effects of CT-MO extract on tunica intima thickness

**Figure 2.**
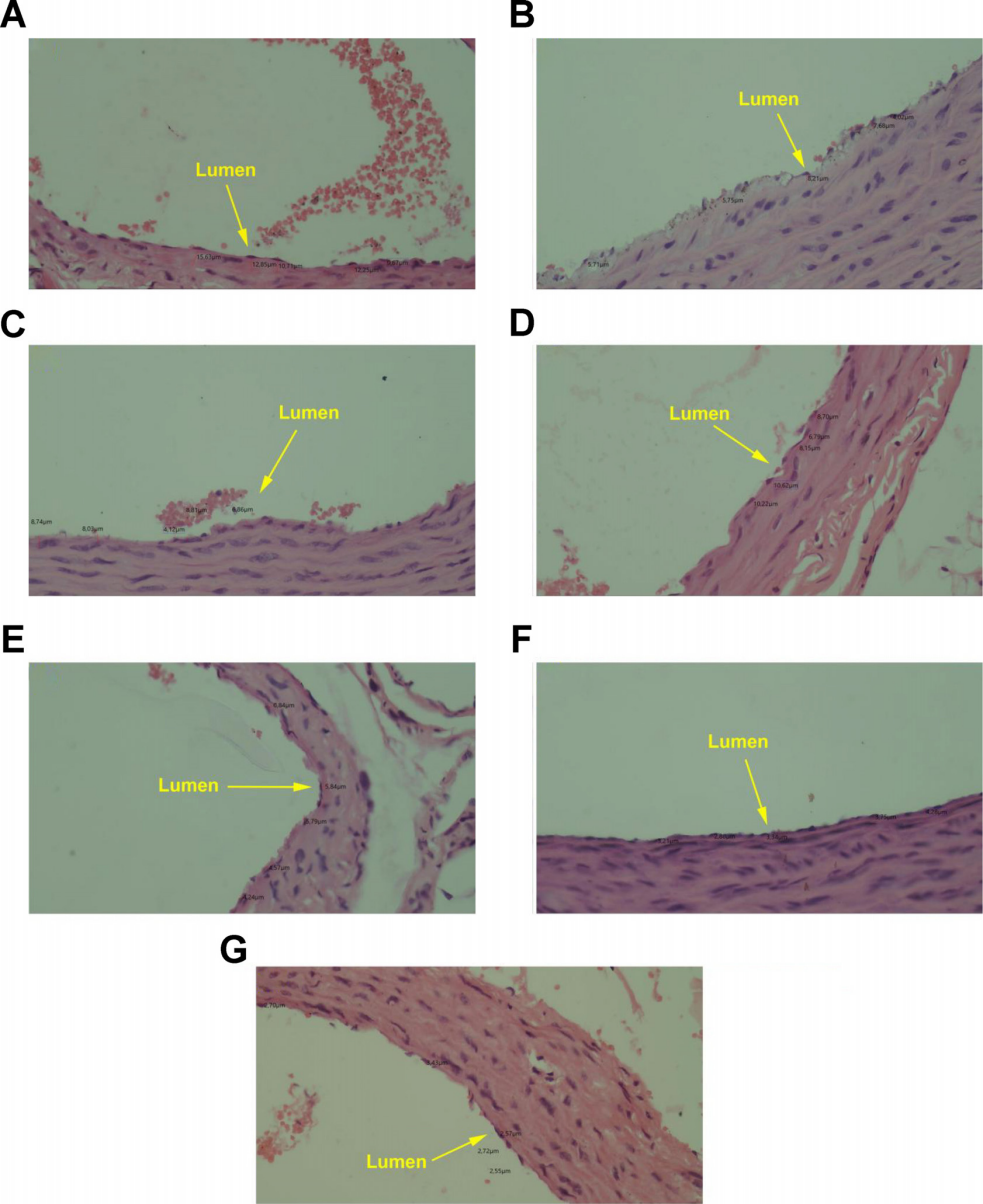
**Histological appearance of the aortic tunica intima across experimental treatment groups.** Representative hematoxylin and eosin-stained thoracic aorta sections showing tunica intima morphology in (A) DC, (B) CT600, (C) MO600, (D) CT-MO200, (E) CT-MO400, (F) CT-MO600, and (G) NC groups. The DC group shows pronounced tunica intima thickening and an irregular luminal surface following HFHC diet and PTU induction. Single-extract treatment with CT600 and MO600 shows partial preservation of the intimal structure, whereas CT-MO treatment demonstrates dose-dependent attenuation of intimal thickening. The CT-MO600 group shows the most preserved aortic morphology among treated groups, with a thin and more regular tunica intima resembling the NC group. Yellow arrows indicate the arterial lumen, and measurement markers indicate tunica intima thickness. Abbreviations: CT, *Clitoria ternatea*; CT-MO, *Clitoria ternatea–Moringa oleifera*; DC, disease control; H&E, hematoxylin and eosin; HFHC, high-fat and high-cholesterol; MO, *Moringa oleifera*; NC, negative control; PTU, propylthiouracil.

Tunica intima thickness varied significantly among groups (*F* ═ 23.503, *P* < 0.001; η^2^ ═ 0.834), indicating a substantial treatment effect (Table 4). Microscopic examination of the aortic wall illustrated the HFHC diet’s impact on vascular structure. In the NC group, the tunica intima appeared thin, smooth, and intact, with a well-organized endothelial layer. Conversely, the DC group exhibited marked pathological changes, characterized by a noticeably thickened and irregular intimal space, indicative of lipid accumulation and cellular infiltration (Figure 2). Treatment with single extracts (CT600 and MO600) and combined CT-MO extracts demonstrated varying degrees of structural preservation, with the CT-MO600 group showing superior preservation of vascular structure. Quantitative analysis confirmed these histological observations. The HFHC diet induced a significant increase in tunica intima thickness in the DC group (12.22 ± 2.28 µm) compared to the NC group (2.78 ± 0.32 µm) (Figure 1E). According to Tukey’s HSD test, the CT-MO600 group (3.42 ± 0.52 µm) exhibited substantial attenuation of intimal thickening, showing no significant difference from the healthy NC group. This structural improvement is consistent with previous biochemical findings, suggesting that the 600 mg/kg BW combination dose may contribute to the preservation of vascular structural features.

### Correlation and multivariate analysis of inflammatory biomarkers and tunica intima thickness

Pearson correlation analysis revealed strong positive correlations between inflammatory biomarkers and tunica intima thickness (Table 5). VCAM-1 exhibited the strongest correlation with tunica intima thickness (*r* ═ 0.919, *P* < 0.001), followed by IL-6 (*r* ═ 0.874, *P* < 0.001), ICAM-1 (*r* ═ 0.866, *P* < 0.001), and CD40 (*r* ═ 0.860, *P* < 0.001). Exploratory multivariate linear regression analysis indicated that VCAM-1 remained significantly associated with tunica intima thickness after adjusting for IL-6, ICAM-1, and CD40 (*B* ═ 0.133, 95% CI: 0.036–0.230, *p* ═ 0.009), whereas the other biomarkers were not statistically significant in the adjusted model. The multivariate regression model demonstrated a high coefficient of determination (R^2^ ═ 0.845).

**Table 5:** Correlation and multivariate regression analysis of inflammatory biomarkers predicting tunica intima thickness

**Biomarker**	**Correlation coefficient**	***P* value correlation**	**Regression B**	**95% CI**	***P* value regression**
VCAM-1	0.919	<0.001	0.133	0.036–0.230	0.009
IL-6	0.874	<0.001	–0.005	–0.069–0.059	0.880
ICAM-1	0.866	<0.001	0.055	–0.327–0.436	0.771
CD40	0.860	<0.001	–0.259	–1.474–0.956	0.666

Multivariate regression model: R^2^ ═ 0.845. Abbreviations: CD40, cluster of differentiation 40; CI, confidence interval; ICAM-1, intercellular adhesion molecule-1; IL-6, interleukin-6; VCAM-1, vascular cell adhesion molecule-1.

## Discussion

The progression of diet-induced atherosclerosis is characterized by sustained endothelial activation and structural remodeling of the arterial wall. The current findings suggest that modulation of inflammatory mediators can concurrently attenuate the expression of adhesion molecules and limit tunica intima thickening, indicating a coordinated effect on both molecular and morphological components of vascular injury. Quantitative phytochemical profiling revealed differential enrichment of secondary metabolites among the single extracts. MO exhibited higher tannin and total phenolic contents, whereas CT contained relatively higher flavonoid levels. This distribution aligns with previous reports identifying MO leaves as abundant sources of phenolic acids and tannins, and CT flowers as rich in flavonoid-based pigments [15, 22, 33]. Importantly, the CT–MO (25:75) formulation yielded the highest flavonoid concentration. The observed vascular protective effects in this formulation may be attributed to its higher flavonoid content; however, the present study did not directly evaluate quantitative correlations between flavonoid levels and inflammatory biomarkers. The effects observed in the CT–MO (25:75) formulation may also reflect complex interactions among multiple phytochemical constituents rather than the activity of a single compound. The observation that the CT–MO (25:75) formulation produced stronger biological effects despite not maximizing all phytochemical classes simultaneously may suggest non-linear interactions among bioactive compounds. However, as interaction-specific analyses were not performed, the present study cannot determine whether these effects reflect synergistic, additive, or antagonistic phytochemical interactions. In light of the present findings, the observed reductions in inflammatory biomarkers may be associated with similar mechanisms. Previous studies have indicated that flavonoids may modulate endothelial function via mechanisms that involve the suppression of oxidative stress and regulation of the NF-κB pathway [22, 46]. NF-κB has been implicated in the regulation of inflammatory mediators such as VCAM-1, ICAM-1, IL-6, and CD40, which are associated with leukocyte adhesion and vascular inflammation [46, 47]. Although the present study did not directly evaluate these pathways, the involvement of NF-κB remains speculative and is primarily inferred from existing literature. Therefore, the dose-dependent reductions observed in these biomarkers following CT-MO administration may suggest broad anti-inflammatory activity associated with the extract treatment, although the precise molecular pathways were not directly investigated in this study [47, 48]. Consequently, the observed biological effects may be linked to the phytochemical-rich composition of the CT-MO formulation, including its flavonoid content, as reported in prior studies [48, 49].

The HFHC diet significantly increased levels of VCAM-1 and ICAM-1, indicating endothelial activation and enhanced leukocyte recruitment, as documented in both experimental and clinical studies [24]. A reduction in VCAM-1 and ICAM-1 to levels statistically comparable to those of healthy controls may signify an improvement in endothelial activation related to diet-induced vascular injury. Notably, the highest combination dose produced values statistically indistinguishable from healthy controls, suggesting a reduction in endothelial inflammatory activation linked to diet-induced endothelial dysfunction [9, 10, 15].

Previous research has indicated that IL-6 may play a role in vascular inflammation through the activation of the IL-6/signal transducer and activator of transcription 3 (STAT3) axis and the modulation of adhesion molecule expression. The substantial decrease in IL-6 in the highest-dose CT-MO group may imply a broader suppression of inflammatory activity; however, the current study was not designed to establish a hierarchical relationship among inflammatory biomarkers. Consequently, the observed reductions in IL-6, VCAM-1, and ICAM-1 should be interpreted as parallel endpoint changes rather than direct evidence of causal signaling interactions. These findings suggest that CT-MO may have broader anti-inflammatory effects beyond adhesion molecules, though further mechanistic studies are needed to confirm this hypothesis [20, 50].

Similarly, CD40 is recognized as a key contributor to vascular inflammation during early atherosclerosis, associated with reductions in inflammatory biomarkers to levels comparable to healthy controls, and immune-mediated vascular injury via the CD40–CD40L pathway [19, 51]. Experimental blockade of this pathway has been linked to reduced inflammatory cell activation and enhanced vascular stability. The significant suppression of CD40 expression in the CT–MO600 group may correlate with modulation of immune-related inflammatory activity associated with chronic vascular inflammation; however, direct mechanistic evaluation was not conducted in this study [52]. Interestingly, the reductions in CD40 levels observed in both the CT600 and CT–MO600 groups were relatively similar, suggesting partially overlapping anti-inflammatory mechanisms between CT and MO rather than fully additive effects on the CD40-related pathway.

Beyond circulating biomarkers, this study demonstrates structural vascular protection. Tunica intima thickening is a hallmark of early atherosclerotic remodeling, indicative of lipid deposition and inflammatory infiltration [20, 23]. The significant reduction in tunica intima thickness in the CT-MO600 group, alongside inflammatory biomarker levels approaching those of healthy controls, suggests that biochemical suppression of inflammation is associated with measurable morphological preservation. This parallel improvement indicates a relationship between reduced inflammatory biomarkers and enhanced vascular structural outcomes [15, 22]. The integration of molecular biomarkers with histological assessment is a key strength of this study, as many previous investigations have focused exclusively on circulating markers without evaluating structural arterial remodeling. By demonstrating concordant reductions in VCAM-1, ICAM-1, IL-6, CD40, and tunica intima thickness, the findings support the potential vascular protective effects of the phytochemical-rich CT-MO extract across multiple inflammatory and structural parameters. However, the current histological evaluation was primarily limited to tunica intima thickness measurements and routine H&E observation, which may not fully capture subtle residual histopathological abnormalities or cellular alterations [13, 20].

Several limitations of this study warrant acknowledgment. First, the relatively small sample size (*n* ═ 5 per group), while consistent with exploratory animal study designs, may limit statistical power and increase the risk of Type II error. Additionally, this study did not include a pharmacological positive control, such as statins or standard anti-inflammatory agents, limiting direct comparisons between the efficacy of the CT-MO extract and established therapies. Second, the combined use of the HFHC diet and PTU may introduce potential confounding effects, as PTU-induced hypothyroidism independently affects lipid metabolism and vascular function. While this approach was employed to accelerate atherosclerotic induction, caution is warranted in interpreting the findings. Furthermore, the 45-day experimental duration may not fully encapsulate the chronic and progressive nature of atherosclerosis. This study also focused exclusively on male Wistar rats, which may limit the generalizability of the findings due to potential sex-related differences in inflammatory and metabolic responses that could influence disease progression and treatment outcomes. Third, the mechanistic interpretation of this study remains indirect; inflammatory pathways such as NF-κB and potential antioxidant activity were not directly assessed through molecular analyses, and oxidative stress markers, including malondialdehyde (MDA), superoxide dismutase (SOD), and catalase, were not evaluated. Thus, the proposed mechanisms should be considered exploratory and primarily based on previous literature rather than definitive causal evidence. Fourth, phytochemical characterization was limited to UV–Vis spectrophotometric analysis, which provides only estimates of total phenolic, flavonoid, and tannin contents without precise identification and quantification of individual bioactive compounds.

Future studies employing advanced analytical techniques such as high-performance liquid chromatography (HPLC) or liquid chromatography–mass spectrometry (LC-MS) are needed for more comprehensive phytochemical characterization. Additionally, histological evaluation was limited to tunica intima thickness measurements using routine H&E staining in five microscopic fields per section, which may not fully represent regional vascular heterogeneity and microscopic sampling variability. This study also did not utilize a factorial or interaction-based experimental design; therefore, synergistic effects between CT and MO could not be conclusively determined. Although the CT-MO (25:75) formulation exhibited the highest estimated flavonoid content along with the most pronounced biological effects, direct statistical correlations between flavonoid concentration and reductions in inflammatory biomarkers were not assessed. Thus, causal attribution of the observed vascular protective effects cannot be conclusively established. Finally, the translation of findings from animal models to human pathophysiology should be approached with caution.

## Conclusion

The combined extracts of *Clitoria ternatea* and *Moringa oleifera* significantly attenuated endothelial activation and tunica intima thickening in a diet-induced early atherosclerosis model. The CT-MO (25:75) formulation exhibited the highest flavonoid content and demonstrated the most pronounced reductions among the tested extract groups. At 600 mg/kg BW, the combined extract significantly reduced levels of VCAM-1, ICAM-1, IL-6, and CD40, and markedly attenuated tunica intima thickening. Correlation and exploratory multivariate analyses further revealed significant associations between inflammatory biomarkers and vascular structural remodeling, with VCAM-1 showing the strongest independent association with tunica intima thickness. These findings support the potential of CT-MO extract as a candidate natural strategy for mitigating endothelial inflammation and early vascular remodeling.

## Data Availability

The datasets generated and analyzed during the current study are available from the corresponding author upon reasonable request and will be made publicly available upon article acceptance.
